# Novel Dimethylmethylene‐Bridged Triphenylamine‐PDI Acceptor for Bulk‐Heterojunction Organic Solar Cells

**DOI:** 10.1002/advs.201700110

**Published:** 2017-06-22

**Authors:** Yu Xiong, Bo Wu, Xiaoyan Zheng, Zheng Zhao, Ping Deng, Ming Lin, Benzhong Tang, Beng S. Ong

**Affiliations:** ^1^ Research Centre of Excellence for Organic Electronics Institute of Creativity and Department of Chemistry Hong Kong Baptist University Hong Kong; ^2^ HKUST Shenzhen Research Institute No. 9 Yuexing 1st RD, Hi‐tech Park Nanshan Shenzhen 518057 P. R. China; ^3^ Department of Chemistry The Hong Kong University of Science and Technology Clear Water Bay Hong Kong; ^4^ Agency for Science, Technology and Research Institute of Materials Research and Engineering 2 Fusionopolis Way 138634 Singapore

**Keywords:** nonfullerene acceptors, organic solar cells, perylene diimide, triphenylamine

## Abstract

A novel, star‐shaped electron acceptor, DMTPA‐PDI_3_, derived from a planar dimethylmethylene‐bridged triphenylamine core with three acetylene‐linked perylene diimide (PDI) units is developed as a nonfullerene acceptor for organic solar cells (OSCs). DMTPA‐PDI_3_ manifests significantly reduced intramolecular twisting, enabling sufficient system‐wide π‐electron delocalization leading to broadened spectral absorption and raised lowest unoccupied molecular orbital level. As a result, higher and more balanced hole and electron transport properties are observed. Active layers for OSCs comprising DMTPA‐PDI_3_ acceptor and PBT7‐Th donor exhibit suppressed intermolecular aggregation, giving rise to uniform nanophase network formation. These OSC devices have afforded respectably high power‐conversion efficiency of about 5%.

Bulk heterojunction (BHJ) organic solar cells (OSCs) with solution‐processed active layers comprising an electron donor and an electron acceptor represent an attractive approach for low‐cost solar energy harvesting and conversion to electricity.^1^, ^2^, ^3^, ^4^ Fullerene derivatives (e.g., [6,6]‐phenyl‐C_61_/C_71_‐butyric acid methyl ester, PC_61_BM/PC_71_BM)^5^, ^6^ have generally been the popular electron acceptors used in OSCs by virtue of, among others, their high electron mobility and isotropic charge transport.^7^, ^8^ However, fullerene derivatives have several drawbacks such as limited spectral absorption, relatively low lowest unoccupied molecular orbital (LUMO) energy level, restricted optoelectronic tunability, poor morphology stability, and high costs due to low synthetic yields and tedious purification, especially for the higher performing C70 derivatives. For these reasons, small molecular^9^, ^10^, ^11^, ^12^, ^13^, ^14^, ^15^ and polymeric^16^, ^17^, ^18^, ^19^, ^20^ nonfullerene acceptors with wider and stronger spectral absorptions, wider tunable energy levels and structural features, have been actively explored as fullerene replacements for OSCs.

In recent years, we have witnessed rapid development of nonfullerene acceptors for OSCs,^21^, ^22^ and perylene diimide (PDI)‐based derivatives have surfaced as appealing nonfullerene acceptor designs.^23^ Advantages of PDI derivatives include relatively high electron mobility, good spectral absorptivity, excellent thermal and photochemical stability, and ease in structural modification for optoelectronic properties. However, unlike the ball‐shape fullerene, PDI core structure has a relatively flat molecular geometry, and thus exhibits a strong tendency to form large aggregates during film formation. This is detrimental to exciton dissociation, and would lead to low power conversion efficiency (PCE) of OSCs. Accordingly, acceptor design strategies incorporating twisted PDI moieties to suppress undesirable molecular aggregation have been explored with varying degrees of success.^23^ In this design approach, PDI units are generally linked at bay or at imide positions via appropriate π‐bridges including thiophene, benzene, tetraphenylethylene, and spirobifluorene, etc.^24^, ^25^, ^26^, ^27^, ^28^ However, most of these PDI‐based acceptors often exhibit severely twisted molecular conformations, hampering intermolecular aggregation and giving rise to amorphous morphologies in their composite layers with common polymer donors. Consequently, low and unbalanced charge transport properties result. For enhanced photovoltaic performance, intramolecular twisting in the acceptor structure should be optimized for nanoscale phase separation in the active layer to enable efficient charge transport.

Triphenylamine (TPA) has been used in constructing star‐shaped molecules for OSCs as it has strong electron donating power and good stabilization effect on hole carriers.^26^, ^29^ The earlier work of Zhan and co‐workers on a TPA star‐shaped acceptor with three PDI units, S(TPA‐PDI), achieved a PCE of over 3% in a nonfullerene OSC device.^26^ However, the severely nonplanar conformation of PDI moieties of this acceptor adversely impacts its charge transport capability while the poor rigidity of TPA core leads to poor thermal and morphological stabilities. These structural deficiencies have essentially limited the photovoltaic properties of S(TPA‐PDI) acceptor. Recently, Yan and co‐workers have shown that reduced intramolecular twisting of PDI moieties improves photovoltaic performance.^30^ In addition, bulky substituents have also been shown to inhibit molecular aggregation of planar acceptor compounds.^31^ It is thus reasonable to anticipate that better photovoltaic performance can be realized through increased structural rigidity and controlled molecular planarity of acceptor compounds. We believe that dimethylmethene bridged triphenylamine (DMTPA), with a rigid flat core structure and sterically repelling peripheral dimethyl substituents, represents an interesting building block for constructing PDI‐based acceptors for OSCs. This would permit tunable molecular coplanarity for modulating molecular aggregation to achieve optimal nanophase separation for efficient charge transport in nonfullerene OSCs.

We report herein our studies on the design, synthesis, and physical and optoelectronic properties of a novel dimethylmethylene‐bridged TPA (DMTPA)‐PDI electron acceptor (DMTPA‐PDI_3_, **Scheme** **1**) for OSCs. DMTPA‐PDI_3_ was constructed from connecting a structurally planar DMTPA core to three PDIs via acetylene bonds. Our design strategy was based on the following considerations: (i) reduced twisting of PDI moieties expected as they are appositely spaced apart and that intramolecular charge‐transfer (ICT) interactions between structurally conjugated PDI acceptor moieties and DMTPA donor core would encourage molecular planarization; (ii) substantial π‐electronic delocalization from DMTPA donor core to PDI acceptor moieties would enhance charge transport property and broaden spectral absorption for solar energy harvesting; (iii) ICT interaction would lead to higher LUMO level, thus improving open‐circuit voltage (*V*_oc_); and (iv) dimethyl substituents would increase molecular solubility and hamper excessive intermolecular aggregation from steric interference. Using a solution‐processed active layer composition of DMTPA‐PDI_3_ as acceptor and PBT7‐Th^31^ as donor in nonfullerene OSC devices, we have been able to achieve PCEs of about 5%. This is significantly higher than that of S(TPA‐PDI) acceptor with a nonplanar TPA core,^26^ and is comparable to many BHJ‐OSCs with PDI‐based acceptors.^23^, ^32^, ^33^, ^34^, ^35^, ^36^, ^37^ These results have clearly demonstrated the greater utility of planar DMTPA as a core structure for designing efficient electron acceptors as fullerene replacement for OSCs.

**Scheme 1:**
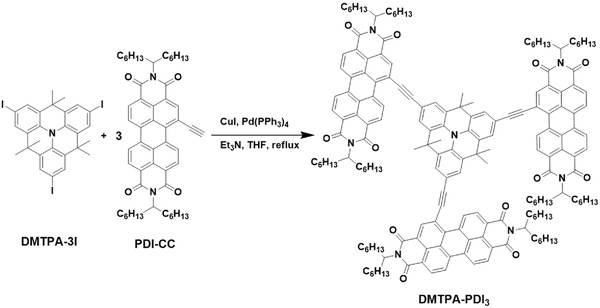
Synthetic route for DMTPA‐PDI_3_.

The synthetic route for DMTPA‐PDI_3_ is outlined in Scheme 1. The ethynyl‐functionalized PDI (PDI‐CC) and triiodo‐functionalized DMTPA (DMTPA‐3I) intermediates were all synthesized in accordance with the reported procedures.^38^, ^39^ Coupling of one equivalent of DMTPA‐3I and three equivalent of PDI‐CC via Sonogashira coupling reaction afforded DMTPA‐PDI_3_ acceptor in moderate yields. All the intermediates and final product were characterized by ^1^H NMR, ^13^C NMR spectroscopy, and high resolution mass spectrometry. DMTPA‐PDI_3_ acceptor was readily soluble in common organic solvents such as toluene, dichloromethane, and chlorobenzene, etc., implicating good solution processibility. Thermogravimetric analysis of DMTPA‐PDI_3_ revealed excellent thermal stability with 5% weight loss at 420 °C (Figure S1, Supporting Information).

The solution (dichloromethane) and thin‐film UV–vis absorption spectra of DMTPA‐PDI_3_ acceptor (**Figure** **1**a) displayed similar absorption profiles with three vibronic peaks in the wavelength range of 450–550 nm and a broad absorption band between 550 and 750 nm which was attributable to the existence of ICT. This was in sharp contrast to only one absorption peak observed for S(TPA‐PDI) acceptor with a nonplanar TPA core.^26^ These experimental results conformed to a higher molecular planarity and greater π‐conjugation system of DMTPA‐PDI_3_ as a consequence of incorporation of a planar DMTPA core and the intervening acetylene spacers. From solution to thin film, the ICT band also exhibited a bathochromic shift of about 25 nm, suggesting extended of π‐conjugation arising from forced molecular planarity in the solid state. However, this small bathochromic shift also implicated weak intermolecular interactions in the solid state. The optical bandgap of DMTPA‐PDI_3_ acceptor, estimated from the onset of thin‐film spectral absorption, was 1.67 eV, which was lower than that reported for S(TPA‐PDI) acceptor.^26^ The electrochemical properties of DMTPA‐PDI_3_ acceptor were examined by cyclic voltammetry (CV) as a thin film deposited on the electrode. As illustrated in Figure 1b, the CV curves exhibited two reversible reduction waves and one reversible oxidation wave. The HOMO and LUMO energy levels were determined to be, respectively, −5.36 and −3.74 eV from the half‐wave oxidation and reduction potentials using Fc/Fc^+^ (−4.8 eV) as standard reference. The energy gap calculated from CV curves was 1.62 eV, which was very close to the optical bandgap of 1.67 eV.

**Figure 1:**
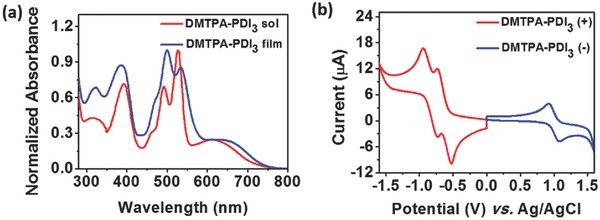
a) Normalized solution (dichloromethane) and thin‐film UV–vis absorption spectra of DMTPA‐PDI_3_. b) Cyclic voltammogram of DMTPA‐PDI_3_ thin film in CH_3_CN/0.1 m *^n^*Bu_4_NPF_6_ at a scan rate of 100 mV s^−1^.

To gain further insights into the electronic properties of DMTPA‐PDI_3_, we performed density functional theory (DFT) geometric optimization for DMTPA‐PDI_3_ at the B3LYP/6‐31G* level by Gaussian 09. The electron density distribution of HOMO and LUMO orbitals of DMTPA‐PDI_3_ are shown in **Figure** **2**a, revealing that the electron density of HOMO was mainly localized on the DMTPA core with a slight delocalization to the PDI moieties through the acetylene bonds. On the other hand, the electron density of LUMO was distributed over only two of the PDI units, suggesting that one of the PDI units was twisted more severely than the other two, thus practically losing its coplanarity or π‐conjugation with the flat DMTPA core. The calculated HOMO and LUMO energy levels were −5.48 and −3.52 eV, respectively, which corresponded very well with the experimental values from CV measurements. Figure 2b shows the top‐view and side‐view of optimized molecular geometry of DMTPA‐PDI_3_ by VMD program.^40^ Two lightly twisted PDI moieties at dihedral angles of, respectively, 15.3° and 18.9° with the central core, and one significantly twisted PDI unit at a dihedral angle of 26.3° were observed. The near‐coplanarity of two PDIs with the central core is rendered possible by a greater spacial freedom of PDI moieties accorded by the intervening acetylene bridges as well as forced coplanarization induced by ICT interactions. This has transformed DMTPA‐PDI_3_ into a substantially π‐conjugated electronic system conductive to charge transport. On the other hand, the significantly twisted PDI moiety and dimethyl substituents on the DMTPA core have served as effective deterrence to strong intermolecular aggregation, thus making nanoscale phase separation of DMTPA‐PDI_3_ feasible for enhanced photovoltaic performance.

**Figure 2:**
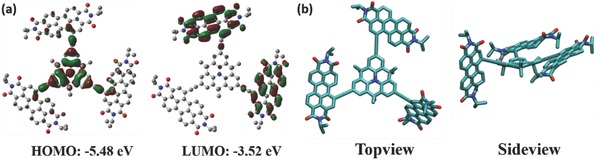
a) HOMO and LUMO electron density contours of optimized DMTPA‐PDI_3_ at B3LYP/6‐31G* level by DFT (top view). b) Top view and side view of optimized DMTPA‐PDI_3_ geometry.

BHJ‐OSC devices with solution‐processed active layers were fabricated using an inverted OSC configuration of ITO/ZnO/Al/PTB7‐Th:DMTPA‐PDI_3_/MoO_3_/Ag with DMTPA‐PDI_3_ as acceptor and PTB7‐Th as donor. The current‐voltage (*J*−*V*) characteristics of the experimental devices are depicted in **Figure** **3**a while other photovoltaic properties, *V*_oc_, short‐circuit current (*J*_sc_), fill factor (FF), and PCE with varying donor/acceptor (D/A) weight ratios and solvent additives are summarized in **Table** **1**.

**Figure 3:**
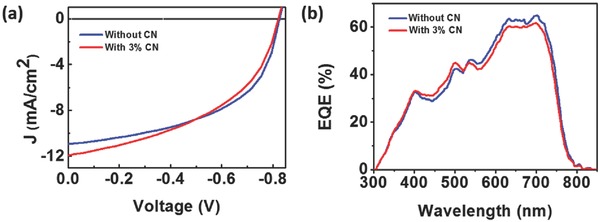
a) *J*–*V* characteristics; b) EQE spectrum for inverted OSC structure of ITO/ZnO/Al/PTB7‐Th:DMTPA‐PDI_3_/MoO_3_/Ag.

**Table 1:** Photovoltaic performances of OSCs based on PTB7‐Th:DMTPA‐PDI_3_ under the illumination of AM 1.5G, 100 mW cm^−2^

PTB7‐Th:DMTPA‐PDI_3_ [w/w]	*V*_oc_ [V]	*J*_sc_ [mA cm^−2^]	FF [%]	PCEa) [%]	PCE_max_ [%]
1:1	0.848 ± 0.003	9.21 ± 0.10	54.94 ± 1.38	4.32 ± 0.07	4.39
1:1.5	0.846 ± 0.002	9.80 ± 0.11	47.81 ± 0.93	3.95 ± 0.08	4.03
1.5:1	0.826 ± 0.003	11.95 ± 0.10	47.82 ± 1.69	4.79 ± 0.09	4.88
2:1	0.855 ± 0.001	7.26 ± 0.18	48.73 ± 0.56	3.04 ± 0.06	3.10
1.5:1 (3% DIO)	0.832 ± 0.005	6.48 ± 0.19	44.01 ± 0.87	2.37 ± 0.06	2.43
1.5:1 (3% CN)	0.829 ± 0.003	11.27 ± 0.27	51.46 ± 0.78	4.81 ± 0.1	4.91

^a)^ The average PCE value was calculated from twelve devices for each condition.

Experimental OSC devices incorporating active layer compositions of PTB7‐Th:DMTPA‐PDI_3_ with weight ratios ranging from 1:1 to 2:1 were fabricated. Under these conditions, the best performing device with a PTB7‐Th:DMTPA‐PDI_3_ ratio of 1.5:1.0 achieved a PCE of 4.88% (Table 1). Using this optimal D/A weight ratio of 1.5:1.0, we investigated the effects of solvent additives, 1,8‐diiodooctane (DIO) and 1‐chloronaphthalene (CN) on photovoltaic performance. While addition of DIO led to poorer photovoltaic performance, 3 vol% of CN was found to further improve the PCE to 4.91%. The observed opposite effects of solvent additives on OSC performance might be attributed to higher solubility of DMTPA‐PDI_3_ in CN. The external quantum efficiency (EQE) spectrum of the device with an active layer composition of PTB7‐Th:DMTPA‐PDI_3_ (1.5:1.0 by weight; 3 vol% CN) displayed a broad photovoltaic response from 300 to 800 nm, in which the maximum EQE value was found to be over 60% (Figure 3b). It is worthy of note that the shape of the EQE spectrum follows very well with the spectral absorptions of active layer (Figure 5a), suggesting that both PTB7‐Th donor and DMTPA‐PDI_3_ acceptor in the active layer contributed to solar energy harvesting and thus photocurrent generation.

As the morphology of active layers have decisive influence over the charge transport and photoelectrical properties of OSCs, we thus conducted atomic force microscopy (AFM) for a composite thin film of DMTPA‐PDI_3_ and PTB7‐Th (1.0:1.5 ratio by weight) processed with 3% of CN additive (active layer composition utilized in our subsequent OSC evaluation) to study its morphology. As reflected in the AFM images (**Figure** **4**a), the solution‐processed composite film exhibited smooth and uniform surface morphology with the root‐mean‐square (RMS) roughness of 2.16 nm. Only nanodomains below 50 nm in size could be visibly observed from the AFM phase image. This smooth nanoscale film morphology demonstrated the efficacy of DMTPA‐PDI_3_ in restraining intermolecular aggregation, and effectively hampering microdomain formation. The observed uniform nanodomain network should be particularly beneficial for exciton dissociation and charge transport in the active layer for OSCs.

**Figure 4:**
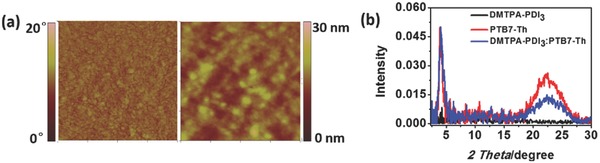
a) AFM images (1 µm × 1 µm) of PTB7‐Th:DMTPA‐PDI_3_ (1.5:1, w/w) thin films (left: phase image; right: height image); b) XRD patterns of DMTPA‐PDI_3_, PTB7‐Th, and PTB7‐Th:DMTPA‐PDI_3_ (1.5:1, w/w) thin films.

To further examine the molecular ordering characteristics of donor and acceptor in the thin film state, three sample films each cast from chlorobenzene solutions of acceptor only, donor only, and acceptor and donor, were subjected to X‐ray diffraction (XRD) characterization using a Bruker D8 Discovery with general area detection diffraction system and a Cu Kα radiation excited at 40 kV and 40 mA. The incoming X‐ray was scanned at a grazing angle of between 0.5° and 2.5°, and the diffractions were recorded by the area detector. Under these conditions, no diffractions were detected in the acceptor‐only film, while those containing donor only, and acceptor and donor showed similar distinct diffractions at 2θ = 21.6°–21.8° and 3.9°–4.0°, arising respectively from lamellar and π–π stacking orderings of PBT7‐Th (Figure 4b). When normalized at the primary interlayer diffraction peak, we could clearly observe the reduction of π–π stacking intensity of PBT7‐Th in the presence of DMTPA‐PDI_3_ acceptor. This reduction in π–π diffraction intensity may be attributed to the suppression of π–π stacking of PBT7‐Th by intermolecular CT interaction between PBT7‐Th donor and DMTPA‐PDI_3_ acceptor. This may have directly contributed to effective suppression of microdomain formation, leading to the observed nanophase network in the composite layer. Nonetheless, over suppression of molecular π–π stacking may disrupt charge transport pathways leading to adverse impacts on overall charge carrier transport properties.

The spectral absorption and emission of active layer composite film (PTB7‐Th:DMTPA‐PDI_3_, 1.5:1.0, w/w) were carefully studied to further understand the photoelectrical performance of DMTPA‐PDI_3_‐based OSCs. As shown in **Figure** **5**a, the active layer displayed strong and broad absorption covering the 300–800 nm spectral region, suggesting a reasonable efficiency in harvesting visible to near infrared solar energy. Furthermore, while both PTB7‐Th donor and DMTPA‐PDI_3_ acceptor each exhibited strong photoluminescence effects at respectively 810 and 780 nm, these photoluminescence were completely quenched when they were mixed together in the composite layer (Figure 5b). This would indicate potentially efficient exciton dissociation in the composite film of this nature for current generation.

**Figure 5:**
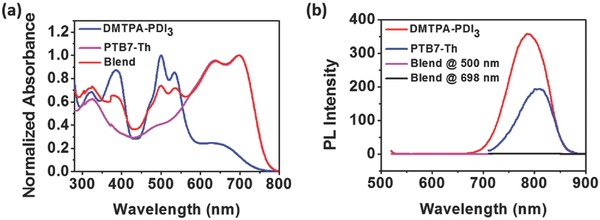
a) Normalized thin‐film UV–vis absorption spectra of DMTPA‐PDI_3_, PTB7‐Th, and PTB7‐Th:DMTPA‐PDI_3_ (1.5:1.0, w/w); b) thin‐film photoluminescence spectra of DMTPA‐PDI_3_ (excited at 500 nm), PTB7‐Th (excited at 698 nm), and PTB7‐Th:DMTPA‐PDI_3_ (1.5:1.0, w/w) (excited at 500 and 698 nm).

The charge transport phenomenon in the optimal PTB7‐Th:DMTPA‐PDI_3_ (1.5:1.0 by weight) active layer composition processed with 3 vol% of CN additive was studied by space charge limited current technique with ITO/PEDOT:PSS/PTB7‐Th:DMTPA‐PDI_3_/MoO_3_/Ag and ITO/ZnO/PTB7‐Th:DMTPA‐PDI_3_/ZnO/Ag as respective device structures for hole‐ and electron‐only measurements. For these devices, a more balanced hole and electron mobility of, respectively, 6.89 × 10^−4^ and 1.98 × 10^−4^ cm^2^ V^−1^ s^−1^ were obtained, in contrast to the low and significantly unbalanced hole and electron mobility of respectively 7.14 × 10^−4^ and 2.32 × 10^−5^ cm^2^ V^−1^ s^−1^ for those devices using S(TPA‐PDI) acceptor.^26^ The higher and more balanced electron and hole mobility in the DMTPA‐PDI_3_‐based devices, which were beneficial to exciton dissociation and charge transport processes, were likely rendered possible by the reduced intramolecular twisting in DMTPA‐PDI_3_.

In summary, a novel, star‐shaped electron acceptor, DMTPA‐PDI_3_, derived from a planar DMTPA core with three acetylene‐linked PDIs was developed for nonfullerene OSCs. DMTPA‐PDI_3_ exhibited a unique quasi‐3D “shallow‐bowl” molecular geometry with significantly reduced intramolecular twisting. As a result of relative molecular planarization from greater degrees of spacial freedom for PDI moieties, DMTPA‐PDI_3_ manifested extensive π‐electron delocalization over the whole molecular system, providing higher and more balanced hole and electron transport properties. Relative molecular planarization of DMTPA‐PDI_3_ also led to efficient ICT interactions which broadened spectral absorption and raised the LUMO level. The quasi‐3D molecular geometry likewise facilitated weak intermolecular aggregation, enabling nanophase network formation which was beneficial to exciton extraction and charge transport. All these attributes had combined to render DMTPA‐PDI_3_ a reasonably efficient acceptor material for use in nonfullerene BHJ‐OSCs. A respectably high PCE of about 5% was obtained. Our results show that DMTPA represents a versatile planar core structure for building potentially efficient electron acceptors to replace fullerene derivatives for OSC applications.

## Experimental Section

*Synthesis of DMTPA‐PDI_3_*: A mixture of PDI‐CC (155.8 mg, 0.2 mmol), DMTPA‐3I (37.2 mg, 0.05 mmol), CuI (2.3 mg, 1.2 × 10^−2^ mmol), and Pd(PPh_3_)_4_ (13.9 mg, 1.2 × 10^−2^ mmol) in tetrahydrofuran (10 mL) and triethylamine (10 mL) was heated to reflux and stirred for 24 h under nitrogen atmosphere. After the reaction, the solvent was removed under reduced pressure and the residue was purified by column chromatography on silica gel using hexane‐dichloromethane (1:2) as eluent, affording DMTPA‐PDI_3_ as a purple solid (100.1 mg, 74.2%). ^1^H‐NMR (400 MHz, CDCl_3_, δ): 10.39 (d, 3H), 8.96–8.93 (m, 3H), 8.81–8.61 (m, 15H), 7.85 (s, 6H), 5.22 (m, 6H), 2.31–2.28 (m, 12H), 1.90 (m, 30H), 1.33–1.23 (m, 96H), 0.86–0.80 (m, 36H); HRMS (MALDI‐TOF) *m*/*z*: [M + Na^+^] calcd for C_183_H_207_N_7_O_12_Na^+1^, 2719.6306; found, 2719.6275.

*Fabrication and Characterization of BHJ‐OSC Devices*: Inverted OSC devices using an Al‐modified ZnO interlayer with a device configuration of ITO/ZnO (10 nm)/Al (1.2 nm)/PTB7‐Th:DMTPA‐PDI_3_ (90 nm)/MoO_3_ (2.0 nm)/Ag were utilized in the evaluation. Prepatterned ITO coated glass substrates with a sheet resistance of ≈15 Ω square^−1^ were cleaned by ultrasonication sequentially with acetone, ethanol, deionized water, and isopropanol each for 10 min. A layer of 10 nm thick ZnO, serving as an electron extraction layer was spin coated onto ITO/glass in a N_2_‐purged glove‐box with O_2_ and H_2_O levels below 0.1 ppm. Subsequently, about 1.2 nm thick Al was deposited onto the ZnO/ITO surface by thermal evaporation in a vacuum chamber with a base pressure of <1.0 × 10^−4^ Pa at evaporation rate of 0.1 Å s^−1^. The Al‐modified ZnO/ITO substrates were then transferred to an adjacent glove box connected to the evaporator with O_2_ and H_2_O levels <0.1 ppm. An active layer solution of PTB7‐Th and DMTPA‐PDI_3_ with appropriate weight ratio in hot chlorobenzene was spin coated onto the substrates. Devices with an active area of 3.0 mm × 3.0 mm were used for evaluation under AM 1.5G illumination at 100 mW cm^−2^ (SAN‐EI Electric XEC‐301S solar simulator). The intensity of solar simulator was calibrated using a silicon reference cell (with KG‐5 filter) to minimize the spectral mismatch.

## Conflict of Interest

The authors declare no conflict of interest.

## Supporting information

Supplementary
