# Supplementary material for: Novel Dimethylmethylene‐Bridged Triphenylamine‐PDI Acceptor for Bulk‐Heterojunction Organic Solar Cells
Source: Adv Sci (Weinh). 2017 Jun 22;4(10):1700110. doi: 10.1002/advs.201700110 (PMC5644237; doi:10.1002/advs.201700110)
Supplement: Supplementary file 1 — Supplementary [file ADVS-4-na-s001.pdf]

Copyright WILEY-VCH Verlag GmbH & Co. KGaA, 69469 Weinheim, Germany, 2017.

ADVANCED SCIENCE
Open Access

Supporting Information

for *Adv. Sci.,* DOI: 10.1002/advs.201700110

Novel Dimethylmethylene-Bridged Triphenylamine-PDI Acceptor for Bulk-Heterojunction Organic Solar Cells

*Yu Xiong, Bo Wu, Xiaoyan Zheng, Zheng Zhao, Ping Deng, Ming Lin, Benzhong Tang, and Beng S. Ong**

Supporting Information

Novel Dimethylmethylene-bridged Triphenylamine-PDI Acceptor for Bulk-heterojunction Organic Solar Cells

*Yu Xiong, Bo Wu, Xiaoyan Zheng, Zheng Zhao, Ping Deng, Ming Lin, Benzhong Tang and Beng S. Ong**

Dr. Y. Xiong, Dr. B. Wu, Dr. P. Deng, Prof. B. S. Ong
Research Centre of Excellence, Institute of Creativity and Department of Chemistry, Hong Kong Baptist University, Hong Kong, SAR, China.
E-mail: bong@hkbu.edu.hk
Dr. Y. Xiong, Dr. Z. Zhao, Prof. B. Tang
HKUST Shenzhen Research Institute, No.9 Yuexing 1st RD, Hi-tech Park, Nanshan, Shenzhen 518057, P.R. China
Dr. X. Zheng, Dr. Z. Zhao, Prof. B. Tang
Department of Chemistry, The Hong Kong University of Science and Technology, Clear Water Bay, Hong Kong, SAR, China
Dr. M. Lin
Institute of Materials Research and Engineering, Agency for Science, Technology and Research, 2 Fusionopolis Way, Innovis138634, Singapore

Materials and Instruments: All chemicals were purchased from commercial suppliers unless otherwise noted and used without further purification. All reactions were performed under a nitrogen atmosphere. Diethyl ether, tetrahydrofuran and toluene were distilled from sodium benzophenone. Intermediate PDI-CC was synthesized according to the reported procedures,[1] and DMTPA-3I was synthesized with slight modification to the reported method.[2]

Nuclear magnetic resonance (NMR) spectra were recorded on a Bruker Ultrashield 400 Plus NMR spectrometer. Thermogravimetric analyses (TGA) were conducted on a TA Instruments Q5000IR at a heating rate of 20 °C min^{-1}under nitrogen gas flow. UV-vis spectra were recorded on a PerkinElmer Lambda 20 UV-vis spectrophotometer. Cyclic voltammentry (CV) measurements were carried out in a deoxygenated anhydrous acetonitrile solution on a CHI600 electrochemical workstation. Pt disk was used as the working electrode, $Ag/AgNO_3$ as the reference electrode, Pt wire as the counter electrode, and 0.1 M tetrabutylammonium hexafluorophosphate as the supporting electrolyte, and the measurements were calibrated with ferrocene (Fc) as the internal standard. AFM measurements were performed by using a Scanning Probe Microscope Dimension 3100 in tapping mode. All film samples were spin-casted onto ITO/ZnO substrates. XRD was carried out on a Bruker D8 Discovery with General Area Detector Diffraction System with a Cu Kα radiation excited at 40 kV and 40 mA. The incoming X-ray was scanned at the grazing angle between 0.5-2.5 degree.

***Theoretical Calculation*:** Theoretical calculations for DMTPA-PDI_3 were performed by Gaussian 09 program[3] using Density Functional Theory (DFT). To simplify computations and enhance computational efficiency, the N-hexylheptyl group of DMTPA-PDI_3 was replaced with a simple N-methyl group, and geometric structural optimization was performed at the B3LYP/6-31G* level, yielding optimized geometric structures to obtain the corresponding electron density.

***Hole- and Electron-only Devices*:** The electron and hole mobilities were evaluated using space charge limited current (SCLC) method using electron- and hole-only devices with respective device architectures of ITO/PEDOT:PSS/PTB7-Th:DMTPA-PDI_3 (140 nm) /MoO_3/Ag and ITO/ZnO/PTB7-Th:DMTPA-PDI_3 (140 nm)/ZnO/Ag. The current-voltage curves were obtained and fitted to a space charge limited form described by:

$$J = (8/9)\ \varepsilon_r \varepsilon_0 \mu\ (V^2/L^3) \qquad (1)$$

where ε_0 is the permittivity of free space, ε_r is the relative permittivity of the material, μ is the electron mobility, V is the voltage drop across the device and L is the thickness of the film. For most polymers, ε_r is around 3.

Figure S1. TGA plot of DMTPA-PDI_3 at a heating rate of 20 °C min^{-1} under N_2 atmosphere.

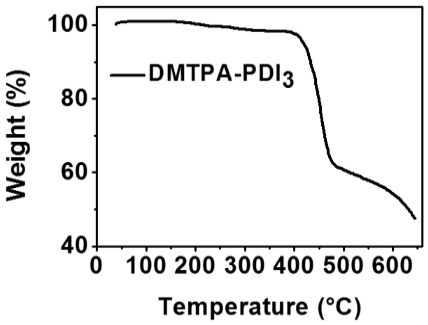

Figure S2. X-ray diffraction pattern of active layer PTB7-Th:DMTPA-PDI_3 (1.5:1 weight ratio, 3% CN).

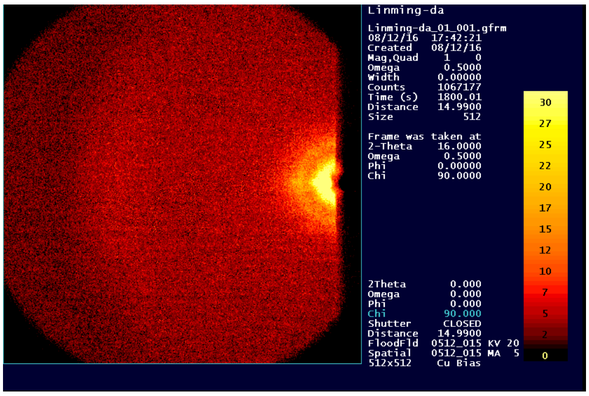

Figure S3. X-ray diffraction pattern of acceptor DMTPA-PDI_3.

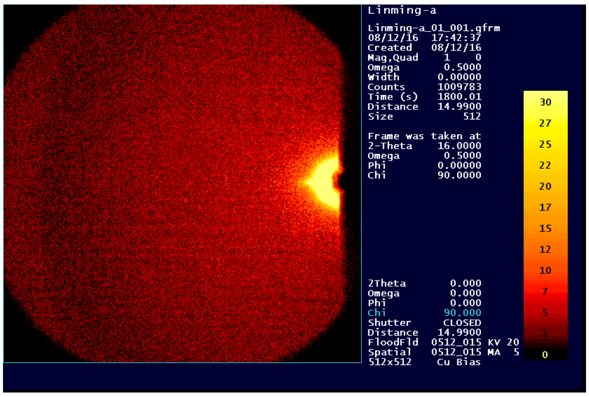

Figure S4. X-ray diffraction pattern of donor PTB7-Th.

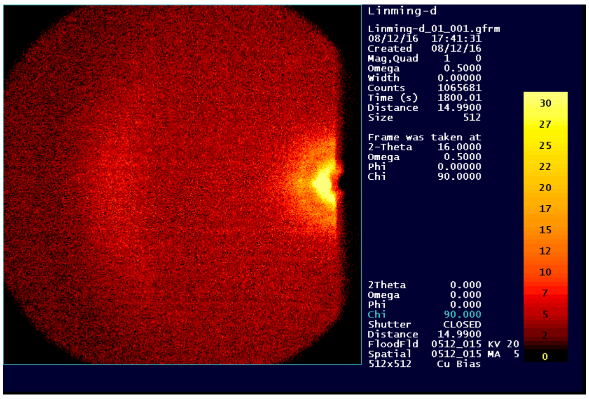

Figure S5. $J_{1/2}$-V characteristics of the hole and electron-only devices

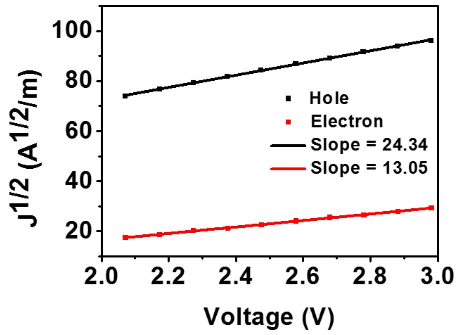

Figure S6. ^{1}H NMR of compound **DMTPA-PDI$_3$**

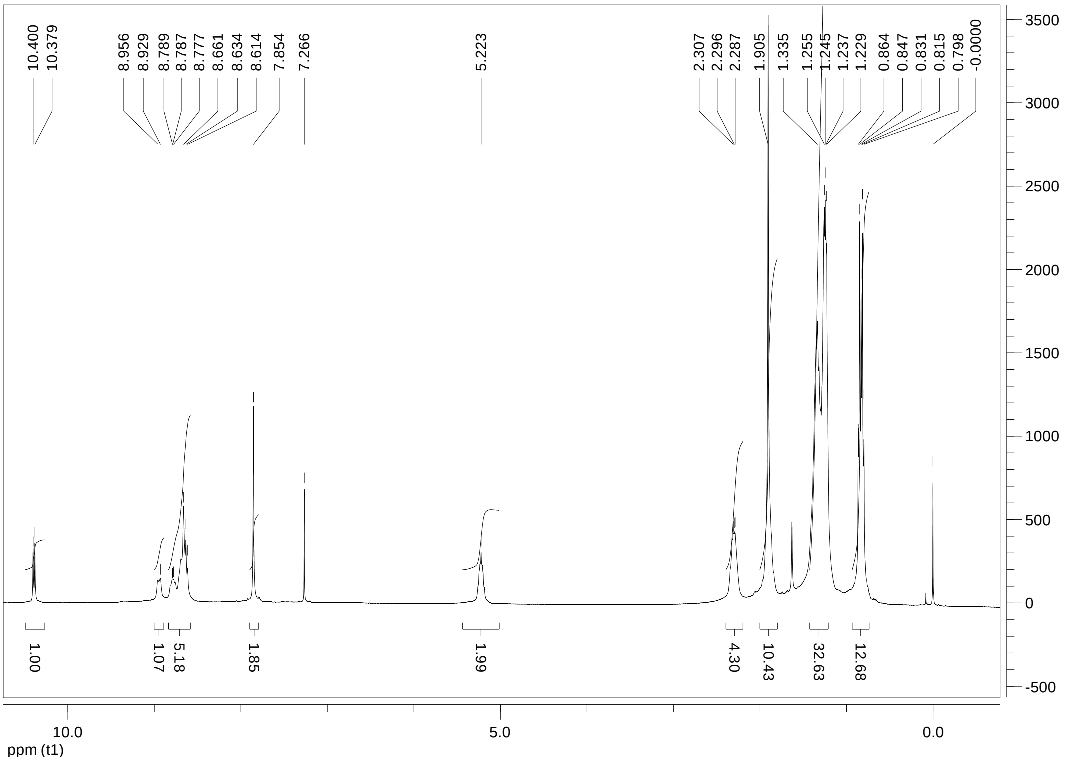

Figure S7. ^{13}C NMR of compound **DMTPA-PDI$_3$**

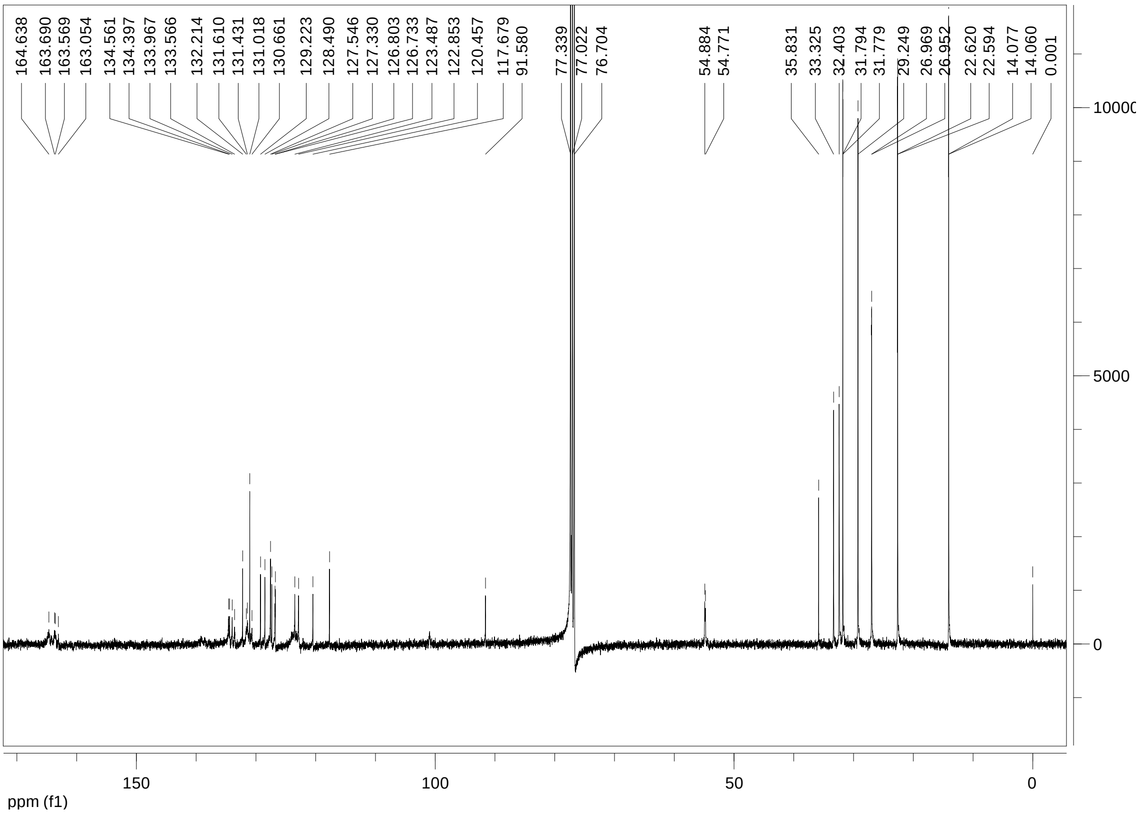

Figure S8. HR-MS (MALDI-TOF) mass spectrum of **DMTPA-PDI$_3$**

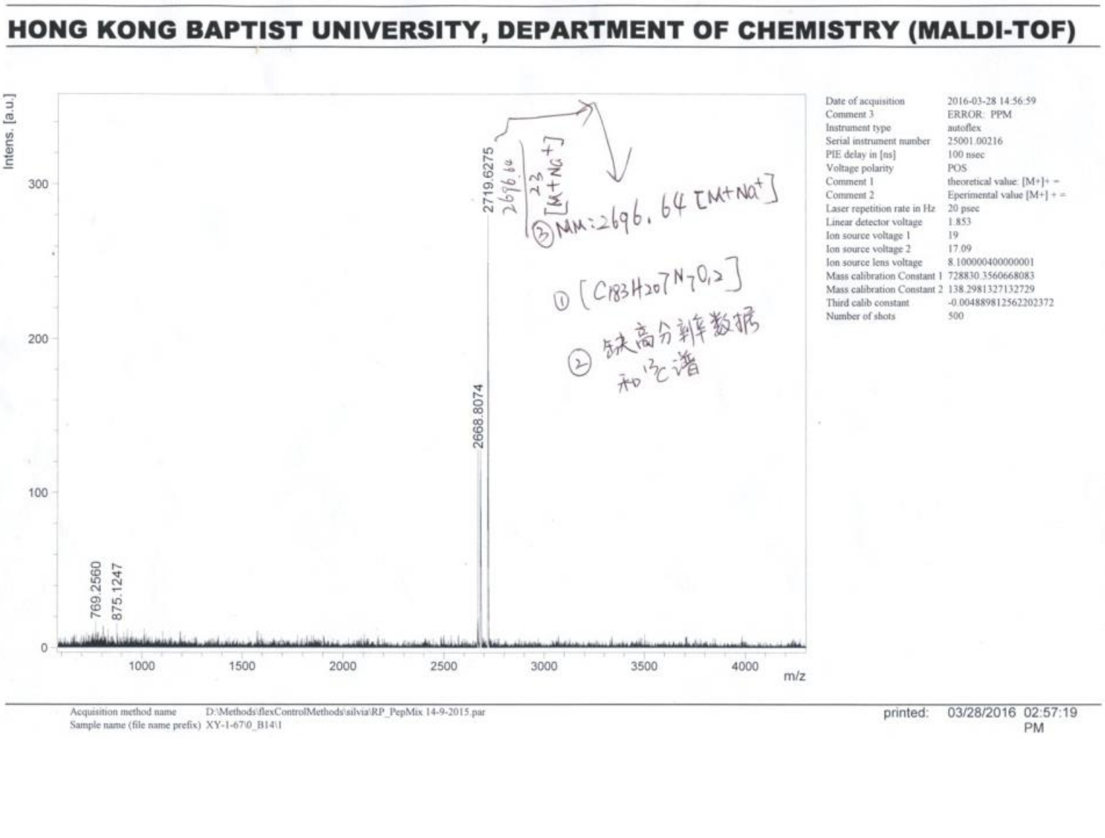

References

[1] Q. Yan, D. Zhao, *Org. Lett.* **2009**, *11*, 3426.

[2] Z. Fang, V. Chellappan, R. D. Webster, L. Ke, T. Zhang, B. Liu, Y-H. Lai, *J. Mater. Chem.* **2012**, *22*, 15397.

[3] M. J. Frisch, G. W. Trucks, H. B. Schlegel, G. E. Scuseria, M. A. Robb, J. R. Cheeseman, G. Scalmani, V. Barone, B. Mennucci, G. A. Petersson, H. Nakatsuji, M. L. Caricato, X.; , H. P. Hratchian, A. F. Izmaylov, J. Bloino, G. Zheng, J. L. Sonnenberg, M. Hada, M. Ehara, K. Toyota, R. Fukuda, J. Hasegawa, M. Ishida, T. Nakajima, Y. Honda, O. Kitao, H. Nakai, T. Vreven, J. Montgomery, J. A.; , J. E. Peralta, F. Ogliaro, M. Bearpark, J. J. Heyd, E. Brothers, K. N. Kudin, V. N. Staroverov, R. Kobayashi, J. Normand, K. Raghavachari, A. Rendell, J. C. Burant, S. S. Iyengar, J. Tomasi, M. Cossi, N. Rega, N. J. Millam, M. Klene, J. E. Knox, J. B. Cross, V. Bakken, C. Adamo, J. Jaramillo, R. Gomperts, R. E. Stratmann, O. Yazyev, A. J. Austin, R. Cammi, C. Pomelli, J. W. Ochterski, R. L. Martin, K. Morokuma, V. G. Zakrzewski, G. A. Voth, P. Salvador, J. J. Dannenberg, S. Dapprich, A. D. Daniels, Ö. Farkas, J. B. Foresman, J. V. Ortiz, J. Cioslowski, D. J. Fox, *Gaussian, Inc.*, Wallingford CT, **2009**.
